# Unilateral Intrastriatal 6-Hydroxydopamine Lesion in Mice: A Closer Look into Non-Motor Phenotype and Glial Response

**DOI:** 10.3390/ijms222111530

**Published:** 2021-10-26

**Authors:** Bárbara Mendes-Pinheiro, Carina Soares-Cunha, Ana Marote, Eduardo Loureiro-Campos, Jonas Campos, Sandra Barata-Antunes, Daniela Monteiro-Fernandes, Diogo Santos, Sara Duarte-Silva, Luísa Pinto, António José Salgado

**Affiliations:** 1 Life and Health Sciences Research Institute (ICVS), School of Medicine, Campus de Gualtar, University of Minho, 4710-057 Braga, Portugal; id7153@alunos.uminho.pt (B.M.-P.); carinacunha@med.uminho.pt (C.S.-C.); anamarote@med.uminho.pt (A.M.); id7472@alunos.uminho.pt (E.L.-C.); id9533@alunos.uminho.pt (J.C.); id7405@alunos.uminho.pt (S.B.-A.); id8942@alunos.uminho.pt (D.M.-F.); a77974@alunos.uminho.pt (D.S.); sarasilva@med.uminho.pt (S.D.-S.); luisapinto@med.uminho.pt (L.P.); 2 ICVS/3B’s—PT Government Associate Laboratory, 4805-017 Guimarães, Portugal

**Keywords:** Parkinson’s disease, 6-OHDA, unilateral, striatum, dopaminergic degeneration, motor behavior, non-motor behavior, glial response

## Abstract

Parkinson’s disease (PD) is a prevalent movement disorder characterized by the progressive loss of dopaminergic neurons in substantia nigra pars compacta (SNpc). The 6-hydroxydopamine (6-OHDA) lesion is still one of the most widely used techniques for modeling Parkinson’s disease (PD) in rodents. Despite commonly used in rats, it can be challenging to reproduce a similar lesion in mice. Moreover, there is a lack of characterization of the extent of behavioral deficits and of the neuronal loss/neurotransmitter system in unilateral lesion mouse models. In this study, we present an extensive behavioral and histological characterization of a unilateral intrastriatal 6-OHDA mouse model. Our results indicate significant alterations in balance and fine motor coordination, voluntary locomotion, and in the asymmetry’s degree of forelimb use in 6-OHDA lesioned animals, accompanied by a decrease in self-care and motivational behavior, common features of depressive-like symptomatology. These results were accompanied by a decrease in tyrosine hydroxylase (TH)-labelling and dopamine levels within the nigrostriatal pathway. Additionally, we also identify a marked astrocytic reaction, as well as proliferative and reactive microglia in lesioned areas. These results confirm the use of unilateral intrastriatal 6-OHDA mice for the generation of a mild model of nigrostriatal degeneration and further evidences the recapitulation of key aspects of PD, thereby being suitable for future studies beholding new therapeutical interventions for this disease.

## 1. Introduction

Parkinson’s disease (PD) is the second most common age-related neurodegenerative disorder, affecting around six million people worldwide [1]. Motor symptoms, such as bradykinesia, rest tremor and rigidity, are the core of PD clinical features that develop due to the progressive loss of dopaminergic (DA) neurons in the substantia nigra pars compacta (SNpc) [2]. This neuronal loss is accompanied by the presence of cytoplasmatic inclusions or Lewy bodies (LBs), mainly composed of α-synuclein protein, that possibly propagates between synaptically-connected areas in a prion-like manner [3]. Moreover, non-motor symptoms like sleep disturbances, intestinal dysfunction, cognitive decline, anxiety and depression, have also been increasingly recognized as major determinants of patient’s quality of life and overall disability [4]. The etiology of PD is still unclear, but it is recognized as a multifactorial disease, involving an interplay between aging, genetic predisposition, and environmental factors [5]. After decades of research, no cure or neuroprotective treatment for PD is available. Although there are treatments to reduce the main symptoms and maintain life quality of the patients, none of them are capable to delay or stop the degeneration of DA neurons [6]. Efforts have been made to develop alternative therapeutical approaches, and for that, the use of PD pre-clinical models is a critical step to test new disease-modifying strategies.

Current PD animal models have been classically generated by targeting the nigrostriatal pathway with toxins or genetic interventions that mimic motor deficits [7]. Since its identification more than 50 years ago, 6-hydroxydopamine (6-OHDA) is still widely used to reproduce PD [8]. The compound 6-OHDA is a catecholamine selective neurotoxin, which assesses the cytosol of DA neurons through dopamine (DA) reuptake transporters, leading to mitochondrial respiratory dysfunction and consequently oxidative stress-induced toxicity and neuroinflammation, which ultimately causes cell death [9]. It is known that the model induced by 6-OHDA does not reproduce all PD features, such as the formation of LBs (an important hallmark of the disease), but notwithstanding this limitation, the relative selectivity of 6-OHDA has been behind its attractiveness to model the classic motor PD symptoms. Moreover, compared with other toxins, such as 1-methyl-4-phenyl-1,2,3,6-tetrahydropyridine (MPTP), 6-OHDA produces robust and relatively stable lesions, without spontaneous recovery [10]. Significant differences in 6-OHDA doses, volumes injected and sites/coordinates of injection are found throughout the literature, which have an impact on the size, location, rate of neurotoxin uptake and therefore in dopaminergic cell survival and behavioral outcomes [11]. When injected into the medial forebrain bundle (MFB) or SNpc, 6-OHDA induces a more complete and rapid lesion in the nigrostriatal pathway, with neurons starting to degenerate within 24 h [12]. On the other hand, if delivered into the striatum, 6-OHDA induces a slow protracted retrograde degeneration, and consequently a more progressive and stable depletion of DA neurons. Therefore, this route of administration offers some advantages over the other regions, since a progressive and less extensive lesion is more relevant in the context of PD [12,13]. Additionally, whereas 6-OHDA lesion in rats is well-described, the generation of 6-OHDA mouse models can be quite challenging, since mice are more prone to high post-lesion mortality and weight loss, therefore requiring intense post-operative care and vigilance [11,14]. Nevertheless, the unilateral administration of 6-OHDA can prevent the high mortality rate observed in bilateral lesions in mice, allowing the use of dosages able to induce motor phenotype, without compromising animals’ well-being [14,15,16,17,18]. Indeed, due to their easier genetic manipulation, the establishment of PD mouse models is key for analyzing the increasingly recognized interplay between genetic and environmental factors. Therefore, these models should recapitulate not only PD-associated motor deficits, but also non-motor phenotypes and other histological alterations than nigrostriatal degeneration, such as glial response. Nevertheless, the assessment of non-motor phenotypes in mice is challenging, especially in unilateral lesions, which might explain the few studies exploring this behavioral dimension in 6-OHDA models. The most reported non-motor phenotypes are anxiety- and depressive-like behavior, which may be more directly implicated in the effects of 6-OHDA lesion in the nigrostriatal pathway [4]. For instance, Bonito-Oliva and colleagues showed that a 6-OHDA lesion into the dorsal striatum resulted in impairment of gait dynamics, accompanied by olfactory deficit, and depression- and anxiety-like behavior [19].

Besides behavioral assessment, the inclusion of glial response analysis is also an important readout of PD models. For several years, the function of glial cells in the central nervous system (CNS) was not fully understood, and they were seen only as supporters of neurons, being important in the maintenance of their viability. Today we know that astrocytes and microglia have a variety of functions in both an intact and injured brain [20]. For instance, astrocytes maintain glutamate, ion and water homeostasis, provide growth factors and metabolites to neurons, and have other functions such as energy storage, mitochondria biogenesis, scar formation, defense against oxidative stress, among others [21]. On the other hand, microglia are involved in synaptic organization, control of neuronal excitability, myelin turnover, phagocytic debris removal, or respond to injury by expressing inflammatory cytokines [22]. Thus, their loss or functional alteration may be directly linked to brain diseases. Glial responses in PD are considered to exhibit dual effects in the neurodegenerative process. Although glial cells are essential for CNS homeostasis, and their activation may benefit DA neurons at early PD stages, the continued and excessive activation of glia can be damaging to neurons and contribute to PD pathogenesis and progression [23].

In the present study, we have reproduced the previously described unilateral intrastriatal 6-OHDA mouse model and performed an extensive behavioral characterization, including both motor and non-motor assessments. In addition, we evaluated neurochemical and histological alterations, including glial activation along the nigrostriatal pathway. This unilateral intrastriatal 6-OHDA model induces a mild degeneration, which is reflected not only in significant motor deficits, but also in subtle non-motor alterations. Moreover, this model is marked by a strong glial response, even 14 weeks after lesion. Therefore, it recapitulates both behavioral and histological phenotypes of PD, thereby being relevant for pre-clinical studies of neuroprotective drugs.

## 2. Results

### 2.1. Motor Performance Characterization

**Figure 1:**
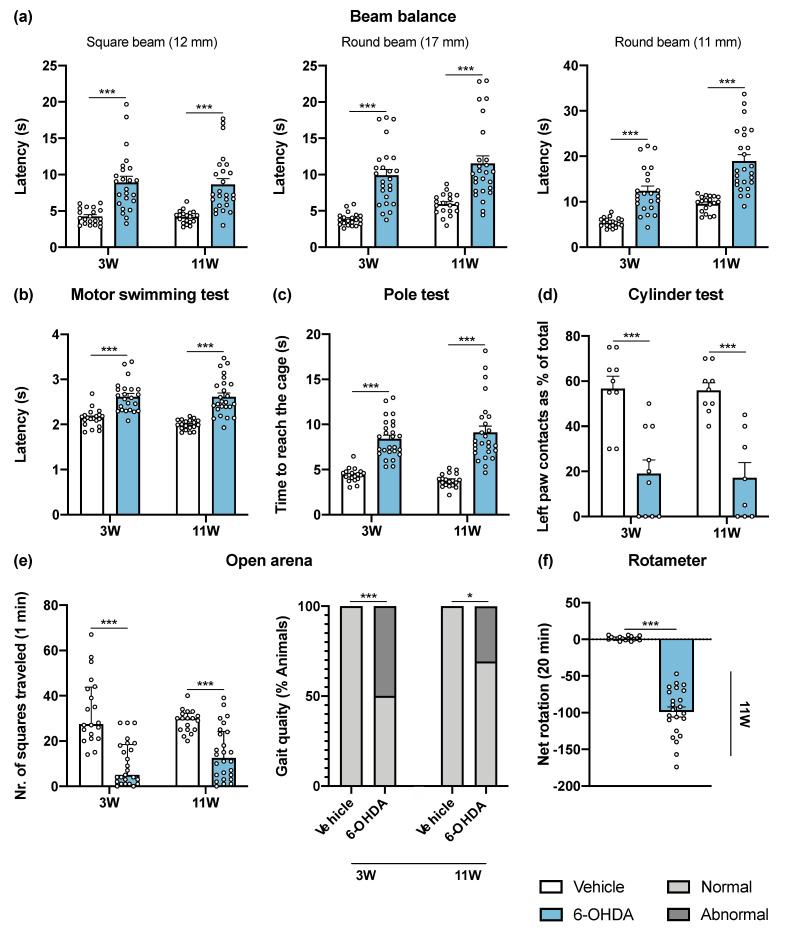
6-OHDA intrastriatal lesion effects on motor behavioral tasks. Overall, all performed tests were found to be sensitive to DA-depleting lesions of the nigrostriatal pathway, 3 and 11 weeks after intrastriatal 6-OHDA injections. 6-OHDA-lesioned mice presented significant impairments in general motor paradigms observed after beam balance (**a**), motor swimming (**b**) and pole (**c**) tests evaluation. The cylinder test (**d**) revealed that 6-OHDA animals use the left (lesioned) forelimb significantly less when compared to the vehicle group (Student’s *t*-test; statistical summary in Table S1; data are presented as mean ± SEM). At the open arena (**e**), it was observed that 6-OHDA injections had a negative impact on the horizontal exploratory activity and animal’s gait quality. The rotameter test (**f**) revealed the success of intrastriatal 6-OHDA lesions with the majority of animals displaying turning behavior after apomorphine administration (Mann–Whitney U test; statistical summary Table S1; data are presented as mean ± IQR). Vehicle n = 19–20, 6-OHDA n = 23–26; except for cylinder test—vehicle n = 8–9, 6-OHDA n = 8–10. * *p* < 0.05, *** *p* < 0.001. Abbreviations: 6-OHDA, 6-hydroxydopamine.

In this work, we performed several motor behavioral tests to characterize a unilateral intrastriatal 6-OHDA PD mouse model, three and eleven weeks after lesion. Data from the behavioral tasks are schemed in Figure 1, while the results of the statistical analyses are presented in Table S1.

The beam balance test was performed to assess balance and fine motor coordination (Figure 1a). Animals were trained in the square beam (12 mm), and on the day of the test, two more beams with a higher level of difficulty were presented to the animals (round beams of 17 mm and 11 mm). 6-OHDA-lesioned animals had a worse performance in all beams, after 3 and 11 weeks post-lesion when compared to the control group. Motor coordination and strength were assessed using the motor swimming test (MST). Presence of the 6-OHDA lesion significantly increased swimming latency of the animals throughout the time course of the experiment (Figure 1b). To monitor locomotor activity, the pole test was also used. 6-OHDA-lesioned animals showed impairments in this test, with 6-OHDA-lesioned animals taking more time to reach the cage (Figure 1c). However, the time that animals took to initiate the movement (latency to turn downward) was similar in both groups (data not shown). The cylinder test measures the spontaneous forelimb use during the exploration of a vertical cylinder wall. Weight-bearing wall touches using contralateral and ipsilateral forelimbs were counted to evaluate the limb use asymmetry after 6-OHDA unilateral lesion. The results showed that 6-OHDA-lesioned animals predominantly use the paw ipsilateral to the lesion (left paw) in exploration of the cylinder walls, compared to the vehicle group (Figure 1d). Spontaneous activity was characterized in terms of horizontal movement in an arena (number of squares crossed), and at the same time the gait of the animals was qualitatively evaluated (scored by the experimenter as normal and abnormal). Horizontal activity was significantly altered by 6-OHDA administration, and some 6-OHDA-lesioned animals showed abnormal gait quality (qualitative assessment, Figure 1e). The rotameter test, using apomorphine, was performed at the end of the motor behavioral assessment to gain insight on functional integrity of the dopaminergic system. Statistical analysis revealed that 6-OHDA lesioned mice displayed significantly more contralateral rotations when compared to the control group (Figure 1f). Rotameter also showed that 88.5% of 6-OHDA group were truly injured after toxin injections. In the animals without turning behavior after apomorphine injections, abnormal involuntary movements were observed, indicating that these animals were also effectively lesioned. Overall, this demonstrates the high success of 6-OHDA injections at the striatum brain region. Overall, all performed tests were found to be sensitive to DA-depleting lesions of the nigrostriatal pathway.

### 2.2. Effect of Intrastriatal 6-OHDA Lesion in Anxiety- and Depressive-like Behavior

Although motor behavioral deficits are commonly reported in 6-OHDA PD models, non-motor features are less frequently addressed and are greatly inconsistent, because of the differences between protocols that can influence the reported outcomes [4]. Therefore, at the end of motor characterization at the last time-point we examined the mice for anxiety- and depressive-like phenotypes.

**Figure 2:**
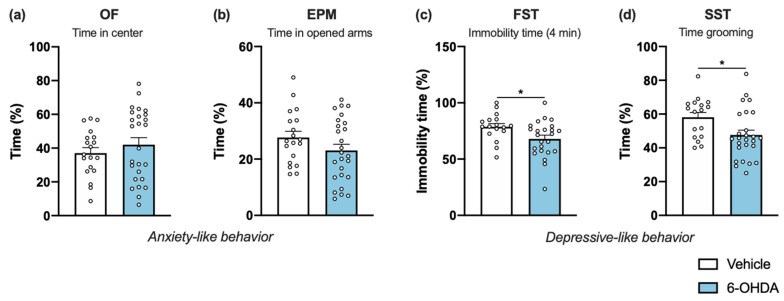
Impact of 6-OHDA intrastriatal lesion on anxiety- and depressive-like behavior. Compared to vehicle, 6-OHDA-lesioned group showed no signs for increased anxiety as seen by the similar time in the center of the OF arena (**a**) and the similar time in the open arms of the EPM (**b**). Regarding depressive-like behavior, in FST (**c**) 6-OHDA-lesioned mice displayed less time immobile but had an increase in time they spent grooming in SST (**d**), when compared to the vehicle group (Student’s *t*-test; statistical summary in Table S1; data are presented as mean ± SEM). Vehicle n = 17–19, 6-OHDA n = 24–26. * *p* < 0.05. Abbreviations: EPM, elevated-plus maze; FST, forced swim test; OF, open field; SST, splash-sucrose test; 6-OHDA, 6-hydroxydopamine.

Mice were examined for anxiety-like behavior in the open field (OF) test. No significant differences were found between groups in the time spent in the anxiogenic center of the arena (Figure 2a). Additionally, no anxious-like signs were confirmed using the elevated-plus maze (EPM) test. During the five-minute test, vehicle and 6-OHDA-lesioned mice spent approximately the same amount of time in opened arms (Figure 2b). The effect produced by striatal 6-OHDA lesion on depressive-like behavior was examined in the forced swim test (FST). We found that 6-OHDA-lesioned animals spent less time immobile in comparison to the vehicle group (Figure 2c), demonstrating that 6-OHDA injections do not have an effect in behavior despair of lesioned animals. On the other hand, the sucrose splash test (SST) paradigm was used to evaluate self-care and motivational behavior, an important feature of depressive-like behavior. Although no differences between groups were found in the latency to initiate grooming (data not shown), 6-OHDA-lesioned mice spent significantly less time grooming after applying a sucrose solution to their coat (Figure 2d). Taken together, these data demonstrated that unilateral striatal 6-OHDA lesion points toward increases in depressive-like behavior, with no indication of alterations in anxiety-like behavior.

### 2.3. Histogical and Neurochemical Characterization of Intrastriatal 6-OHDA Lesion

At the end of the in vivo experiment, animals were divided into two tissue-processing groups: whole brain fixing for subsequent sectioning and immunohistochemistry, or macrodissection of specific brain regions for neurochemical analysis.

**Figure 3:**
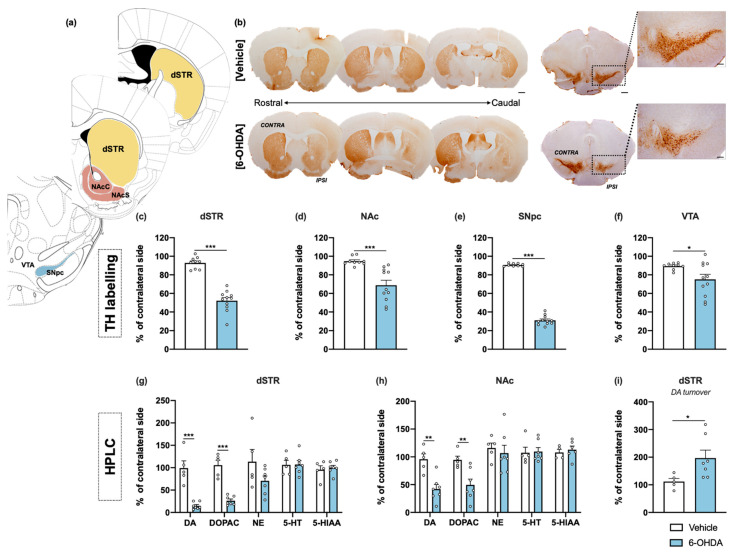
Histological and neurochemical characterization of intrastriatal 6-OHDA lesions. Representative image of the brain regions analyzed (**a**), using coronal sections of the mice’s brains. Brightfield photomicrographs of representative coronal brain sections stained for TH (**b**). Compared to the vehicle group, 6-OHDA animals presented marked reduction of TH+ labelling into the dorsal striatum (**c**) and NAc (**d**), as well as significant loss of TH+ cells in the SNpc (**e**) and VTA (**f**). HPLC quantification, combined with electrochemical detection, showed a significant decrease of DA and its metabolite DOPAC in both dorsal striatum (**g**) and NAc (**h**)**,** after 6-OHDA administration. No differences were found between groups for the monoamines and its derivates (NE, 5-HT, and 5-HIAA), for both regions (**g**,**h**). For the dorsal striatum, the reduced levels of DA were accompanied with an increased DA turnover in this brain region (**i**) (Student’s *t*-test; statistical summary in Table S1; data are presented as mean ± SEM and as % of contralateral side). For histological analysis vehicle n = 9, 6-OHDA n = 11; for HPLC vehicle = 4–5, 6-OHDA n = 6–7. * *p* < 0.05, ** p < 0.01, *** *p* < 0.001. Scale bar = 1 mm. Abbreviations: dSTR, dorsal striatum; DA, dopamine; DOPAC, 3,4-dihydroxyphenylacetic acid; NAcC, nucleus accumbens core; NAcS: nucleus accumbens shell; NE, norepinephrine; SNpc, substantia nigra pars compacta; TH, tyrosine hydroxylase; VTA, ventral tegmental area; 5-HIAA, 5-hydroxyindoleacetic acid; 5-HT, serotonin; 6-OHDA, 6-hydroxydopamine.

For histological assessment, tyrosine hydroxylase (TH) immunohistochemistry in the striatum, SNpc and ventral tegmental area (VTA) was performed. Representative examples of successful lesions are shown in Figure 3b in all these regions. TH+ cells were counted in the SNpc and VTA; and TH+ labelling was measured in the striatum—separated into dorsal striatum and nucleus accumbens (NAc). Both histological analyses in the lesioned hemisphere were compared and presented as percentage of contralateral side. The 6-OHDA injections caused a significant reduction of TH+ labelling in the dorsal striatum on the side of the lesion when compared to the vehicle group (Figure 3c). In the NAc, TH+ labelling was also significantly reduced, although to a lesser extent than dorsal striatal density (Figure 3d). From the results, analysis also revealed statistical differences in the number of TH+ neurons in the SNpc between groups (Figure 3e). Moreover, although intrastriatal lesions mainly affect dopaminergic neurons of the SNpc, they also lead to a decrease in VTA DA neurons, which constitute the mesolimbic pathway and innervate the nucleus accumbens. However, loss of VTA DA neurons does not exceed the 20% that is normally reported in other studies using a similar lesion (Figure 3f) [24].

Regarding neurochemical analysis, high-performance liquid chromatography (HPLC) measurements were performed for specific monoamines and their derivatives in the major projection targets of DA neurons—dorsal striatum and NAc. 6-OHDA animals presented significant decreases of DA and its metabolite 3,4-dihydroxyphenylacetic acid (DOPAC) in both areas (Figure 3g,h). In parallel with DA reduced levels, we observed an increased turnover of DA in the dorsal striatum (Figure 3i), indicated by the increased ratio of DA metabolite (DOPAC) to DA, which was not verified in the NAc region. No differences were found between groups for norepinephrine, serotonin (5-HT) and its metabolite 5-hydroxyndoleacetic acid (5-HIAA), for both areas (Figure 3g,h). The raw data regarding histological and neurochemical characterization is presented in detail in Supplementary Figure S1. Overall, the results indicate 6-OHDA induces a mild neurotoxic effect, mainly limited to the dorsal striatum and its dopaminergic afferents from the SNpc.

### 2.4. Glial Cell Reactivity in Lesioned Areas

**Figure 4:**
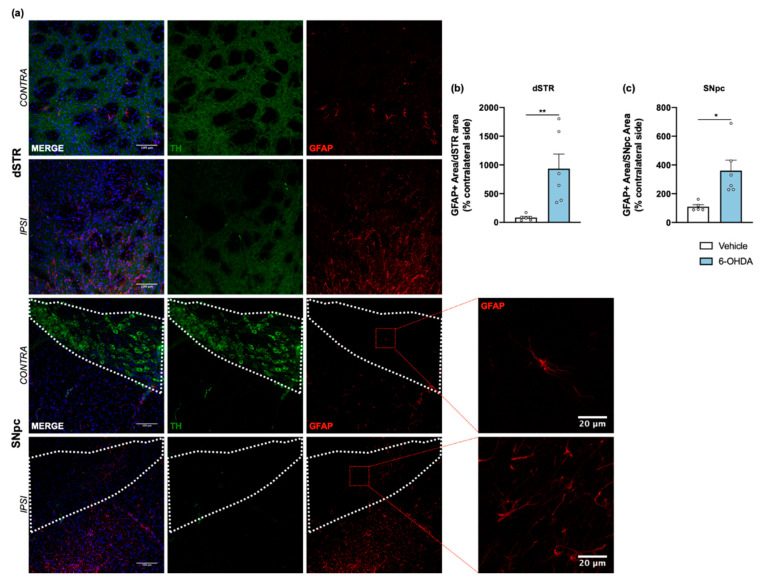
Astroglial reaction after 6-OHDA intrastriatal lesion. Confocal representative photomicrographs (**a**) showing labelling of DA neurons (green) and of astrocytic cells (red) from the dorsal striatum and SNpc, of contra- and ipsilateral sides of 6-OHDA-lesioned mice. Quantification of area coverage by GFAP+ staining showed a significant astrocytic reaction in the ipsilateral lesioned areas (**b**,**c**) of 6-OHDA mice. Area coverage was acquired from each 20× magnification image, and for SNpc, the area was delineated considering the dopaminergic neurons labelled for TH (Student’s *t*-test; statistical summary in Table S1; data are presented as mean ± SEM and as % of contralateral side). Vehicle n = 4–5, 6-OHDA n = 6–7 (four slices/animal). * *p* < 0.05, ** *p* < 0.01. Scale bars = 100 μm and 20 μm. Abbreviations: DA, dopamine; dSTR, dorsal striatum; GFAP, glial fibrillary acidic protein; SNpc, substantia nigra pars compacta; TH, tyrosine hydroxylase; 6-OHDA, 6-hydroxydopamine.

**Figure 5:**
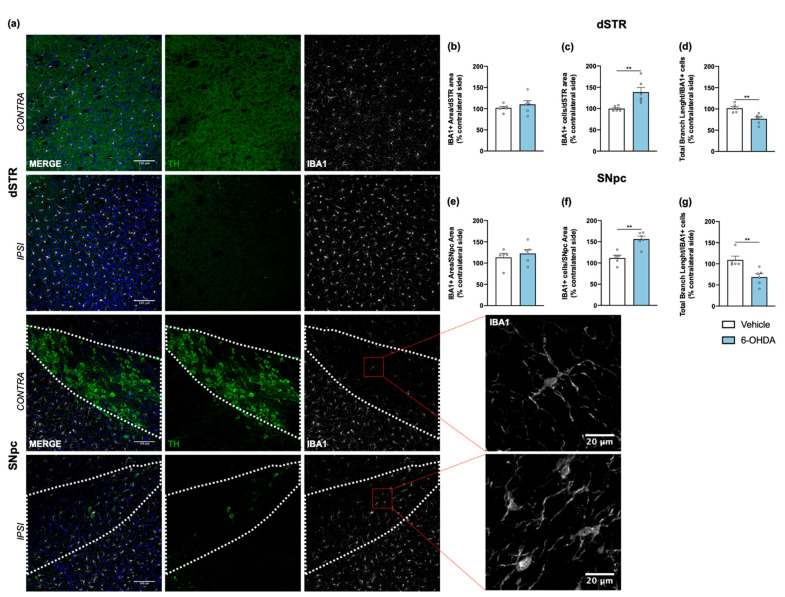
Microglial response after 6-OHDA intrastriatal lesion. Confocal representative photomicrographs (**a**) showing labelling of DA neurons (green) and of microglial cells (grey) from the dorsal striatum and SNpc, of contra- and ipsilateral sides of 6-OHDA-lesioned mice. No differences were found between groups after quantification of area coverage by IBA1+ staining in the dorsal striatum (**b**) and SNpc (**e**). A significant increase of IBA1+ cells (**c**,**f**), as well as a significant decrease of IBA1+ total branch length (**d**,**g**) in the ipsilateral areas of 6-OHDA group was observed, indicating the presence of proliferative and activated microglia. All measurements were performed in acquired 20× magnification images, and for SNpc, the area was delineated considering the dopaminergic neurons labelled for TH (Student’s *t*-test; statistical summary in Table S1; data are presented as mean ± SEM and as % of contralateral side). Vehicle n = 4–5, 6-OHDA n = 6–7 (four slices/animal). ** *p* < 0.01. Scale bars = 100 μm and 20 μm. Abbreviations: DA, dopamine; dSTR, dorsal striatum; SNpc, substantia nigra pars compacta; TH, tyrosine hydroxylase; 6-OHDA, 6-hydroxydopamine.

Tissue sections from dorsal striatum and SNpc were stained with anti-GFAP antibody to visualize astrocytic cells (Figure 4a). Results showed a significant increase of astrocyte reactivity in 6-OHDA lesioned mice when compared to the vehicle group for both areas (after measuring the area occupied by GFAP+ cells and its processes; Figure 4b,c). A similar analysis was also performed for microglia, through IBA1 staining (Figure 5a). Contrary to astrocytes, no differences were found between groups in IBA1+ area (Figure 5b,e). However, we observed a significant increase of IBA1+ cells in both dorsal striatum and SNpc (Figure 5c,f). Moreover, to understand if IBA1+ cells were more activated (less ramified) in 6-OHDA lesioned mice, microglial branching pattern was evaluated using skeleton analysis (Figure S2). Interestingly, results revealed that the total branch length of IBA1+ cells was significantly decreased after 6-OHDA lesion for both lesioned areas (Figure 5d,g). The raw data regarding glial cell reactivity characterization is presented in detail in Supplementary Figure S3. Altogether, these results reveal a marked astrocytic response in this model, as well as microglial proliferation and activation, that is still present even 14 weeks after 6-OHDA injections.

## 3. Discussion

In this study, we report a complete behavioral, histological, and neurochemical characterization of a unilateral intrastriatal 6-OHDA mouse model, which evidences its value for mimicking a mild degeneration phenotype. Unilateral lesions in the striatum, as the one herein described, are a valuable tool for reproducing a less severe degeneration, since the striatum is a larger brain region than MFB and SNpc, and therefore it is easier to deliver 6-OHDA stereotactically, enhancing the lesion success in mice. Indeed, all animals injected with 6-OHDA were effectively lesioned, as evidenced by the turning behavior in the rotameter test, as well as by the loss of DA neurons in the nigrostriatal pathway revealed by TH staining in the striatum and SNpc, and DA levels quantification in the striatum. Moreover, this type of quantification benefits from the lesion’s unilaterality, as side-specific behaviors, as well as lesioned and intact hemispheres of the brain, can be more easily compared [11]. Finally, in contrast to bilateral 6-OHDA administration, unilateral lesions avoid profound impairments in feeding, drinking and animals’ general health. In our study, the survival rate was greater than 70%, a value that could be further increased using enhanced post-operative care in future studies [25].

Regarding motor phenotype, our assessment revealed significant long-term alterations in general motor performance paradigms (Figure 1), namely deficits in balance and fine motor coordination in the beam balance test, MST and pole test. Impaired forelimb use was also revealed in the cylinder test, which is a standard test to identify unilateral lesions in rats. Spontaneous activity was evaluated in an open arena, revealing compromised horizontal movement and abnormal gait pattern, characterized by dragging of the lower part of the body (walking with the body closer to the floor) and exhibited a backwards positioning of the left (lesioned) hindlimb. These behavioral outcomes are in line with other studies using similar lesions in mice [14,15,17]. Although we did not perform some classical tests used in PD animal models, such as the rotarod and staircase tests, in this work we demonstrate that other tests that normally are used in bilateral lesions (e.g., beam balance, MST and pole tests), are also sensitive to intrastriatal unilateral lesions. Moreover, these tests are more straightforward, without requiring food restriction (e.g., required in the staircase test), especially difficult in mice, and long periods of baseline assessment, and are also less dependent of animals’ motivation to perform the task, a common problem faced in the rotarod test.

Concerning drug induced rotational behavior, which is often used for confirming the lesion success in unilateral models, from the total 26 animals to which 6-OHDA was administered, 23 animals presented turning behavior (i.e., increased number of contralateral rotations) after apomorphine administration, thereby confirming the successful depletion of DA on the lesioned side (Figure 1f). Although, at least in rats, it is described that a denervation beyond 90% is necessary to induce consistent hypersensitivity and apomorphine rotation [26], 0.1 mg/kg dose of apomorphine used in this study induced turning behavior without the development of significant stereotyped behavior. Indeed, our histological and neurochemical analysis disclosed a significant degeneration at the nigrostriatal pathway and striatal DA depletion in 6-OHDA lesioned animals, which was lower than 90% that is usually reported for MFB and SNpc lesions [12]. We observed approximately 40% loss of TH+ labelling in the dorsal striatum and 60% loss of TH+ cells from the SNpc, as well as a less pronounced degeneration in VTA and NAc, which is in line with previous studies [14,15,27]. Of note, although we have used a slightly higher 6-OHDA dosage (18 μg) than usually reported (up to 12 μg), it did not seem to have a major impact on the extent of nigrostriatal degeneration. Interestingly, in the dorsal striatum, we observed that reduced levels of DA were accompanied by an increase in the DOPAC/DA ratio, indicating that remaining DA neurons may present an increased activity. In fact, an increase in DA turnover has been hypothesized as a compensatory mechanism for dopaminergic neuronal loss in early PD stages, which may increase the formation of reactive molecules (e.g., hydrogen peroxide and DA quinone) that arise from DA metabolism, and thus further contribute to dopaminergic loss in the long term, due to the reduced antioxidant capacity of SNpc [28,29,30,31]. Therefore, our study demonstrates that important neurochemical aspects of PD progression can also be recapitulated in this unilateral intrastriatal 6-OHDA model, offering opportunities for further studies of how DA metabolites affect the degenerative process.

In addition to classical motor symptoms, PD patients display incapacitating neuropsychiatric manifestations, such as apathy, depression, and anxiety, which are under-recognized and consequently undertreated [4,32]. Accordingly, these non-motor phenotypes are also less studied in PD animal models, especially in mice [4]. Indeed, to our knowledge, this is the first report on the neuropsychiatric effects of unilateral intrastriatal 6-OHDA injections in mice. Previous studies using bilateral intrastriatal 6-OHDA mouse models have consistently shown significant effects on anxiety- and depressive-like behaviors [4,19]. Nevertheless, our results on the OF and EPM tests show no signs of anxiety-like behavior, even though 6-OHDA lesioned animals present a decrease in the distance travelled in the EPM and OF, as well as an increased time resting, which can confound the interpretation of the results. It is known that locomotor activity in the OF depends not only on motor skills, but also on the motivation to explore a novel arena; two behavioral dimensions in which the striatum is critically involved [33]. The dorsal striatum is essential for motor control and motor learning, whereas the ventral striatum, particularly NAc, is a key structure of emotional and motivation processing, modulating the reward system and acting as the limbic–motor interface [34]. As mentioned above, TH+ labelling in VTA and NAc is significantly affected after 6-OHDA injections. Moreover, the levels of DA and its metabolite DOPAC, measured using HPLC, were also significantly reduced in the NAc of 6-OHDA-lesioned mice (Figure 3h). Therefore, it is possible that the lesion of mesocorticolimbic network may contribute to decrease the drive to explore, as supported by other studies [35,36,37], and thus, have an impact on the performance of the tests herein described. Moreover, previous reports of anxiety-like behavior on bilateral intrastriatal mouse models are observed in animals with a reduced level of motor deficits because of the low dosage of 6-OHDA used, which can also contribute to a better readout on EPM and OF tests. On the other hand, our results on the SST reveal a decreased grooming behavior in 6-OHDA lesioned animals (Figure 2d), which is interpreted as a decrease in self-care and considered similar to motivational deficit and apathetic behavior observed in depressive patients [38]. This result may be explained by the partial, yet significant, degeneration of the VTA, a structure that is involved in emotional activities (e.g., motivation, reward, and addiction), and that is reported to be affected (decreased neuronal number) in severely depressed patients [39,40]. The VTA has also been implicated in PD pathophysiology, though with relative sparing of neurodegeneration, possibly due to the diverse neuronal populations found in VTA, lower expression of DA transporter, and differences in cytosolic DA levels and calcium channel expression [41]. Contrarily to this significant effect on the SST, the FST showed similar latency to immobility between control and lesioned animals (data not shown), and a subtle decrease in the immobility time of 6-OHDA lesioned animals (Figure 2c). This result is not in line with previous reports using bilateral striatal lesions in mice, which showed an increase in the immobility time for 6-OHDA-lesion animals—considered as a measure of behavioral despair [19,25]. However, FST could also be interpreted as a measure of learned-helplessness, a depressive-like paradigm that has been shown to be affected in unilateral intrastriatal 6-OHDA lesions in rats [42].

Finally, since glial activity is an important event of 6-OHDA toxicity [43], the present study also investigated alterations in glial cells within this unilateral intrastriatal 6-OHDA mouse model. Consistently with previous findings [44,45,46,47], we observed a significant increase in the area occupied by GFAP+ cells (specific for astrocytes) in the ipsilateral dorsal striatum and SNpc of 6-OHDA-lesioned animals (Figure 4), as well as a significant increase of IBA1+ cells in lesioned areas, indicative of microglial proliferation, with a significant decrease of IBA1+ total branch length, which suggests an activated phenotype. Of note, this evaluation of glial reactivity was only performed 14 weeks after 6-OHDA administration, whereas previous studies report this assessment at earlier timepoints (up to 6 weeks after 6-OHDA lesion), which may indicate the importance of glial response for 6-OHDA model stability and probably contributes to block behavioral compensation normally observed after moderate neurodegeneration. Indeed, in our study, there was a strong correlation between the number of IBA1+ in SNpc and the loss of TH+ neurons in this region, as well as between IBA1+ cells activation and the loss of dopaminergic fibers in the dorsal striatum (see Supplementary Materials, Figure S4 and Table S2). The specific role of astrocytes and microglia in the context of PD remains unclear, with both the excessive reaction and loss of normal activity of astrocytes being proposed as possible causes of the high vulnerability of the DA neurons. Astrocytes can stimulate or prevent neuronal damage, and the loss of balance could be essential for the onset and progression of PD [47]. An interesting study showed that fluorocitrate, that causes activation, prolonged dysfunction, and death of astrocytes, accelerated neuronal degeneration in SNpc induced by 6-OHDA. Stereological analysis indicated decreased TH+ neuronal density already seven days after combined treatment with 6-OHDA and fluorocitrate, while the effect of 6-OHDA alone was only partial [48]. Furthermore, several postmortem and PET studies revealed the presence of reactive microglia both in the striatum and SNpc, and also the expression of proinflammatory molecules such as IFN-γ, TNF-α and IL-1β [49,50]. The deleterious role of these inflammatory mediators on DA neuron’s survival has been demonstrated in several experimental in vitro and in vivo models. For instance, the toxicity of lipopolysaccharide (LPS) to DA neurons was only observed in vitro in the presence of microglia [51]. In vivo, after 6-OHDA administration, some studies suggest that activation of microglia precedes DA neuronal cell loss [52,53]. These findings, and the fact that the SNpc region is particularly rich in microglia [54], strongly suggest that these cells may play a crucial role in PD pathogenesis. However, it is important to keep in mind that microglial activation is not always detrimental (for example, microglia plays a crucial role in phagocytic removal of cell debris and dead neurons), and its neuroprotective effects in CNS diseases, including PD, have gained special attention in recent years [55].

Overall, the unilateral intrastriatal 6-OHDA mouse model presented in this study highlights several aspects of the PD neurodegenerative process. Our protocol showed to be sensitive to voluntary simple motor behavior tasks and an attractive tool for the behavioral and mechanistic investigation of non-motor symptoms. Nevertheless, the degeneration in the nigrostriatal system may not be sufficient to mimic the still poorly understood neuropathological substrates of non-motor symptomatology in PD patients. Therefore, in the future, animal models should move in the same direction of clinical findings regarding PD pathophysiology. Moreover, in this study, the degeneration in the nigrostriatal pathway was accompanied by a reactive glial response in lesioned areas, which is an important feature of PD neuroinflammation process. In our view, this animal model is a powerful tool, that can be applied to future studies looking at therapeutic interventions.

## 4. Materials and Methods

### 4.1. Animals

A cohort of 55 male and female C57/BL6J mice aged 10–12 weeks (21–28 g) were used in this study. The sample size needed was calculated using the G*Power tool, assuming an effect size of 0.8 (Cohen’s d), with a statistical power of 0.8 and a confidence interval of 95%. Animals were housed in groups of 5–6 animals in filtered-topped polysulfone cages (Techniplast, Buguggiate, Italy), with corncob bedding (Scobis Due, Mucedola, Settimo Milanese, Italy) in a SPF animal facility. All animals were maintained in the following conditions: artificial 12:12 h dark/light cycle with lights on at 08:00 h, room temperature (RT) of 21 ± 1 °C, relative humidity of 50–60%, with food (standard diet 4RF21; Mucedola, Settimo Milanese, Italy) and water ad libitum. All experiments were conducted in compliance with local regulations on animal care and experimentation (European Union Directive 2010/63/EU), and with consent from the Portuguese national authority for animal research, Direção Geral de Alimentação e Veterinária (ID: DGAV 005,454 23/04/2025, Lisbon, Portugal) and Ethical Subcommittee in Life and Health Sciences (SECVS; ID: SECVS-142/2016, University of Minho, Braga, Portugal).

### 4.2. Experimental Design

**Figure 6:**
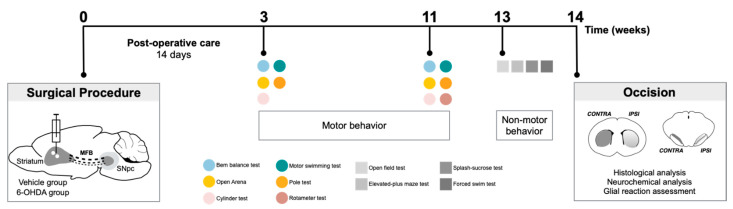
Experimental design. Schematic representation and temporal sequence of performed tasks throughout the experiment.

The sequence of the experiments carried out in this study is graphically outlined in Figure 6. Unilateral lesions in the dopaminergic system were achieved by injecting 6-OHDA at dorso-lateral striatum. Animals underwent surgical procedures for 6-OHDA lesions, and the degree of the lesion was assessed in a series of motor behavioral tests (balance beam, motor swimming test, pole test, spontaneous activity evaluation in open arena, cylinder test, and rotameter test). Behavioral assessment was performed 3 and 11 weeks after lesion surgery to evaluate possible progression of the lesion along time. In the last time-point of analysis, the open field, elevated plus maze, forced-swim test, and sucrose splash test were also conducted to identify the presence of non-motor symptoms in this model. At the end of behavioral assessment, animals were oscised, and brains dissected and processed for TH histological analysis, neurochemical analysis, and evaluation of glial reaction in lesioned areas (these data were analyzed under blinded conditions). In this study, the experimental groups have both males and females, since differences between sexes in terms of motor behavioral phenotypes and histological analysis was not observed (see Supplementary Materials, Figure S3 and Table S2).

### 4.3. Lesion Procedure (6-Hydroxidopamine Injections)

Mice were intraperitoneally (i.p.) anesthetized with ketamine-medetomidine [75 mg/kg; 1 mg/kg] diluted in 0.9% NaCl. Once anesthetized, animals were positioned on a stereotaxic frame (Stoelting, Kiel, WI, USA), and unilateral lesions were achieved by injection of 6-OHDA hydrochloride (H4381, Merck, Darmstadt, Germany) in the right hemisphere using a 10 μL Hamilton syringe (DDBioLaB, Barcelona, Spain). The neurotoxin was used at a concentration of 6 μg/μL (calculated from free base weight) dissolved in a solution of 0.9% NaCl in 0.2 mg/mL ascorbic acid. 6-OHDA was administrated into the dorsolateral striatum (2 × 1.5 μL) at a rate of 300 nl/min, at the following stereotaxic coordinates (relative to bregma, in mm): (i) AP = +1.0, ML = −2.1, DV = −2.9; (ii) AP = +0.3, ML = −2.3, DV = −2.9. After each injection, the needle was left in place there for 5 min for diffusion and to avoid any backflow. The control group was injected in the same conditions with the neurotoxin vehicle. Anesthesia was reversed using atipamezole (1 mg/mL). Animals were randomly assigned into both experimental groups.

### 4.4. Post-Operative Care

Post-operative mortality is generally high in 6-OHDA mice models, and animals can suffer from a variety of complications (Table S3) [11,25]. However, this can be overcome by continuous health assessment and intensive post-operative care after lesion surgery. Thus, health status of each animal was assessed during the first 14 days post-surgery, and mice that reached the human endpoints [56] were euthanized. During the recovery, animals were housed in a warmed room and post-operative care was performed twice a day.

Following surgery, animals received 0.1 mg/kg injections of buprenorphine (i.p.) every 6 h for 5 days to avoid pain, and 5 mg/kg carprofen subcutaneously (s.c.) every 24 h for 3 days. Animals received 1 mL injections (s.c.) of sterile and warm saline twice daily to mitigate dehydration, and if needed, they were fed by hand with providing Nutri-plus Gel (Virbac, Nice, France) into the mouth via 1 mL syringe. Until the animals got used to the energy supplement, s.c. injections of 1 mg/kg/day Duphalyte (Zoetis, Parsippany-Troy Hills, NJ, USA) were also administered. Mice were weighed every day in the first week post-surgery, and then once a week until the end of the experiment (Figure S5). The mortality rate of mice in the 6-OHDA group was 25.7% (9 animals were lost during the experiment).

### 4.5. Behavioral Assessment

Prior to each behavioral test, mice were habituated to the testing room for 20 min. Behavioral tests were conducted in the order outlined in (Figure 6) and as described below.

#### 4.5.1. Beam Balance

This test was performed as previously described [57]. Animals were trained for 3 days on the square beam (12 mm), and at day 4 they were tested on the training beam and on two round beams (17 mm and 11 mm). The time the animal took to cross the beam was registered, and time was discounted whenever the animal stopped. If the animal fell or turned around, this was considered a failed trial. Each animal had the opportunity to fail 2 times on each beam.

#### 4.5.2. Motor Swimming Test (FST)

For MST, mice were trained for 2 consecutive days (3 trials per animal) to traverse a clear Perspex water tank (100 cm long) to a safe black platform at the end. Mice were tested for 3 consecutive days (2 trials/animal), and the latency to cross the water was measured from 60 cm distance. Water temperature was monitored and set to 23 °C using a thermostat [57].

#### 4.5.3. Pole Test

Mice were placed facing up on top of a pole (37.5 cm long) located in their cages. The time the animals took to reach the cage was registered. If the animal fell or did not turn down after 5 min, this was considered a failed trial. Time values were registered on 3 consecutive days (2 trials/animal) [58].

#### 4.5.4. Open Arena

Mice were transferred to a 15-labeled-square arena (55 cm × 33 cm × 18 cm) and the number of squares travelled in the arena for 1 min was counted. Gait quality was also scored as normal or abnormal, by the same experimenter [59].

#### 4.5.5. Cylinder Test

To perform this test, mice were placed individually in a transparent cylinder (11.5 cm diameter) and were video recorded for 5 min to record the laterality of forelimb use of the animals when exploring the cylinder wall. A mirror was placed behind the cylinder at an angle to allow the record of the forelimb movements along 360°. The videos were analyzed in slow motion and frame-by-frame to score the rears, and the number of wall contacts performed independently with the left and the right forepaw were counted to a total number of 20 wall contacts per mice and session. This test was performed during the night cycle when animals are more active to explore [14,60].

#### 4.5.6. Rotameter Test

To estimate the dopaminergic denervation after 6-OHDA injections, the rotameter test using apomorphine was performed. For that, animals received a s.c. injection of 0.1 mg/kg apomorphine hydrochloride (A4393, Merck) dissolved in 1% ascorbic acid, 0.9% NaCl. Each mouse was placed in a glass cylinder, and full turns were counted in the ipsilateral and contralateral directions during 20 min. Data are expressed as total net contralateral rotations (representing the difference between the total number of contralateral and ipsilateral rotations). Mice received 2 apomorphine injections over the preceding days to avoid any potential wind-up effect [61].

#### 4.5.7. Open Field (OF) Test

For the OF test, animals were placed at the center of an illuminated square arena (43.2 cm × 43.2 cm), enclosed by a 30.5 cm high wall. Their movements were tracked for 5 min, using a 16-beam infrared system (MedAssociates Inc., Twin City, GA, USA), and data was analysed using the Activity Monitor software (MedAssociates Inc.). The distance and time at the center/periphery were considered as measurements of anxiety-like behavior [62].

#### 4.5.8. Elevated-Plus Maze (EPM) Test

The EPM apparatus (ENV—560, MedAssociates Inc.) was composed of two opposite open arms (50.8 cm × 10.2 cm) and two closed arms (50.8 cm × 10.2 cm × 40.6 cm) elevated 72.4 cm above the floor and softly illuminated. Each mouse was placed into the center of the maze (10 cm × 10 cm), facing one of the two open arms, and its behavior was video recorded for 5 min. Trials were recorded using an infrared photobeam system, and the percentage of time spent in each of the three compartments (open, close, center) was measured through the EthoVision XT 11.5 tracking system (Ethovision, Noldus Information Technologies, Leesburg, VA, USA). The propensity to avoid the open arms was considered as an index of anxiety [63].

#### 4.5.9. Forced Swim Test (FST)

FST was performed as previously described [64]. For that, each animal was individually placed in glass cylinders filled with water (23 °C; depth 30 cm) during 6 min. All sessions were video-recorded, and the immobility time was measured. Mice were considered immobile when remained passively floating or doing minimal movements need to maintain the head above the water surface. The first 2 min of the trial were considered as a habituation period and the last 4 min as the test period.

#### 4.5.10. Splash-Sucrose Test (SST)

To perform this test, a 10% sucrose solution was sprayed on the dorsal coat of mice in their home cage. The latency to initiate a grooming behavior, as well as the duration of grooming, was recorded during 5 min. The sucrose solution was chosen as a releaser and a persisting factor of grooming due to its viscosity [65].

### 4.6. Histological Assessment

After behavioral assessment, animals were deeply anesthetized with a mixture of ketamine (150 mg/kg) plus medetomidine (0.3 mg/Kg), and a cohort of animals of each group was transcardially perfused with a 4% paraformaldehyde in PBS. Brains were collected and immediately embedded in OCT and frozen at −80 °C for further processing in the cryostat. Coronal sections were then cut at 40 μm and representative sections of striatum and SNpc were selected for free-floating immunohistochemistry to visualize dopaminergic innervation. In brief, the endogenous peroxidases activity was stopped using 3% hydrogen peroxidase for 20 min RT, followed by permeabilization in 0.1% PBS-T for 10 min (3 times) and blockage in PBS 10% newborn calf serum (NBCS) for 2 h. Sections were then incubated with anti-TH from rabbit (1:1000; AB152, Merck) overnight at 4 °C. The next day, sections were incubated with a biotinylated secondary antibody (TP-125-BN, ThermoFisher Scientific, Waltham, MA, USA), followed by strepptavidine-peroxidase solution (TP-125-HR; ThermoFisher Scientific) for 30 min at RT. Antigen revelation was done using 3-3′-diaminobenzidine tetrahydrochloride (DAB; 25 mg DAB in 50 mL Tris–HCl 0.05 M with 12.5 μL H2O2, pH 7.6; D5905, Merck). Sections were mounted on slides, and after 24 h drying in the dark, they were mounted using Entellan (Merck, Darmstadt, Germany).

TH+ labelling in the dorsal striatum and NAc were imaged using brightfield illumination (SZX16, Olympus, Tokyo, Japan) and the mean grey value was measured using the ImageJ software (v1.48, National Institute of Health, Stapleton, NY, USA) as previously described in detail [66], taking into account the anatomic landmarks represented in Figure 3a. Briefly, the measurements are taken from specified ROIs positioned in the specific areas (dorsal striatum and NAc), and the signal from measurements of background (mean intensity from ROIs positioned in cortical areas) is subtracted. TH+ cells in the SNpc and VTA were visualized and counted using a brightfield microscope (BX51, Olympus) equipped with a digital camera (PixeLINK PL-A622, CANIMPEX Enterprises, Zurich, Switzerland). Through the Visiomorph software (v2.12.3.0, Visiopharm, Horsholm, Denmark), the boundaries of the SNpc and VTA areas were drawn, and total TH+ cells were counted in both hemispheres. Data is presented as the percentage (%) of contralateral side (intact side). Raw data relative to histology results are represented in Figure S1a.

### 4.7. Neurochemical Analysis

Macrodissection was performed in a set of animals of each group after decapitation and brain snap-freezing in liquid nitrogen. Brain areas of interest were rapidly dissected using a brain slicer, observing anatomical landmarks. Samples were snap-frozen (dry ice) and stored at −80 °C for further experiments.

Quantification of catecholamine levels in the striatum and nucleus accumbens (NAc) was performed by high-performance liquid chromatography, combined with electrochemical detection (HPLC/EC) using a Gilson instrument (Gilson, Mettmenstetten, Switzerland), fitted with an analytical column (Supleco Supelcosil LC-18 3 mM; flow rate: 1.0 mL/min). Left and right striatum and NAc were weighted and then incubated with 0.2 N perchloric acid, sonicated (5 min on ice) and centrifuged at 5000× *g*. The resulting supernatant was filtered through a Spin-X HPLC column (Costar, ThermoFisher Scientific) to remove debris and 150 μL aliquots were injected into the HPLC system, using a mobile phase of 0.7 M aqueous potassium phosphate (pH 3.0) in 10% methanol, 1-heptanesulfonic acid (222 mg/L) and Na-EDTA (40 mg/L). A standard curve using known concentrations of all monoamines was run each day. Monoamine’s concentration in each side were calculated using the standard curve and normalized to the amount of tissue from which they were extracted. Data is presented as the percentage (%) of contralateral side (intact side). Raw data relative to HPLC results are represented in Figure S1b.

### 4.8. Immunofluroescense and Detection of Glial Cells

For detection of glial fibrillary acidic protein (GFAP) and ionized calcium binding adaptor molecule 1 (Iba-1), representative sections of striatum and SNpc were permeabilized in 0.3% PBS-Triton X-100 (PBS-T) for 10 min (3 times), followed by blockage using 0.3% PBS-T with 10% NBCS for 1 h. Sections were then incubated with the following primary antibodies: anti-TH from mouse (1-1000; MAB318, Merck), anti-GFAP from goat (1-200; ab53554, Abcam, Cambridge, UK) and anti-Iba-1 from rabbit (1-750; 019-19741, Wako, Bellwood, VA, USA), diluted in 3% PBS-T with 2% NBCS overnight at 4 °C. After washing, tissue sections were incubated with the secondary antibodies (Alexa Fluor: 488 donkey anti-mouse, 594 donkey anti-goat and 647 donkey anti-rabbit; ThermoFisher Scientific), diluted in 3% PBS-T for 2 h at RT. Nuclei were stained with 4-6-diamidino-2-phenylindole-dhydrochloride (DAPI, 1:1000; ThermoFisher Scientific) for 10 min at RT. Afterwards, coverslips were mounted on glass slides using PermaFluor (ThermoFisher Scientific).

For image acquisition, two imaging workflows were developed for the scanning of SNpc and striatal areas, respectively. Photomicrographs from triple immunofluorescence stains were acquired with a confocal laser scanning microscope (Olympus FV3000) and are representative of 4 slices per animal. To standardize image acquisition and minimize inter-sample variation, all photos were taken at a sampling speed of 2 μs/pixel and pinhole aperture of 132 μm, using lasers 405 (Blue), 488 (Green), 594 (Red) and 647 (Grey), and scanned with a 3.5 micron step-size over the Z-plan. Striatal images were acquired along the dorsal–ventral and medial–lateral axis, and tiles encompassing the whole SNpc area were imaged using a motorized XY scanning stage equipped with a 20× objective (UPlanSApo NA 0.75). Images had a resolution of 1.257 px/micron and a voxel size of 0.795 × 0.795 μm^3^. Individual acquisition settings for each marker of interest can be found in Table S4.

### 4.9. Image Analyis of Glial Reaction

Photomicrographs containing the four channels were analyzed using a semi-automated workflow on ImageJ software. Measurements of the area occupied by astrocytes (GFAP-positive stain), as well as counts and branching patterns (skeleton analysis) for microglial cells were assessed in both lesioned areas. Images were first processed to produce projections along the *Z*-axis, and ROIs were implemented with the polygon tool to segment the striatal and nigral areas. Automatic and manual intensity-based thresholding was applied to segment microglial and astrocytic cells, respectively. The area occupied by the GFAP staining was measured and normalized against the control from both the SNpc and striatum ROIs. Manual counting of microglial cells (Iba-1-positive cells) was performed using the multi-point tool from the (Cell-Counter) plugin. Microglial branching was assessed with the skeletonize tool after the application of an unsharp mask and the close function. Total branch length was normalized by the number of microglial cells within each image [67]. Data are presented as the percentage (%) of contralateral side (intact side). Raw data relative to glia reaction image analysis results are represented in Figure S3.

### 4.10. Statistical Analysis

Statistical analysis was performed using GraphPad Prism v.8.0c (GraphPad Software, San Diego, CA, USA). A significance level of α = 0.05 was chosen for all the comparisons. The assumption of normality was tested for all continuous variables through evaluation of the frequency distribution histogram, the values of skewness and kurtosis and through the Shapiro–Wilk test. All continuous data are shown as the mean ± SEM. For the comparison of means between two groups, a Student’s *t*-test for independent samples was used, when data was continuous; for discrete data, a Mann–Whitney U test was carried out and data presented as mean ± IQR. A critical value for significance of *p* < 0.05 was used throughout the study. For Supplementary Figures S2 and S3, a Pearson correlation and two-way ANOVA analysis were performed, respectively. Statistical summary of all conducted analysis is presented in Tables S1 and S2.

## Data Availability

Data is contained within the article or Supplementary Material.

## Supplementary Materials

The following are available online at https://www.mdpi.com/article/10.3390/ijms222111530/s1.
