# Supplementary material for: Unilateral Intrastriatal 6-Hydroxydopamine Lesion in Mice: A Closer Look into Non-Motor Phenotype and Glial Response
Source: Int J Mol Sci. 2021 Oct 26;22(21):11530. doi: 10.3390/ijms222111530 (PMC8584172; doi:10.3390/ijms222111530)
Supplement: Supplementary file 1 [file ijms-22-11530-s001.zip › ijms-1413457-supplementary.pdf]

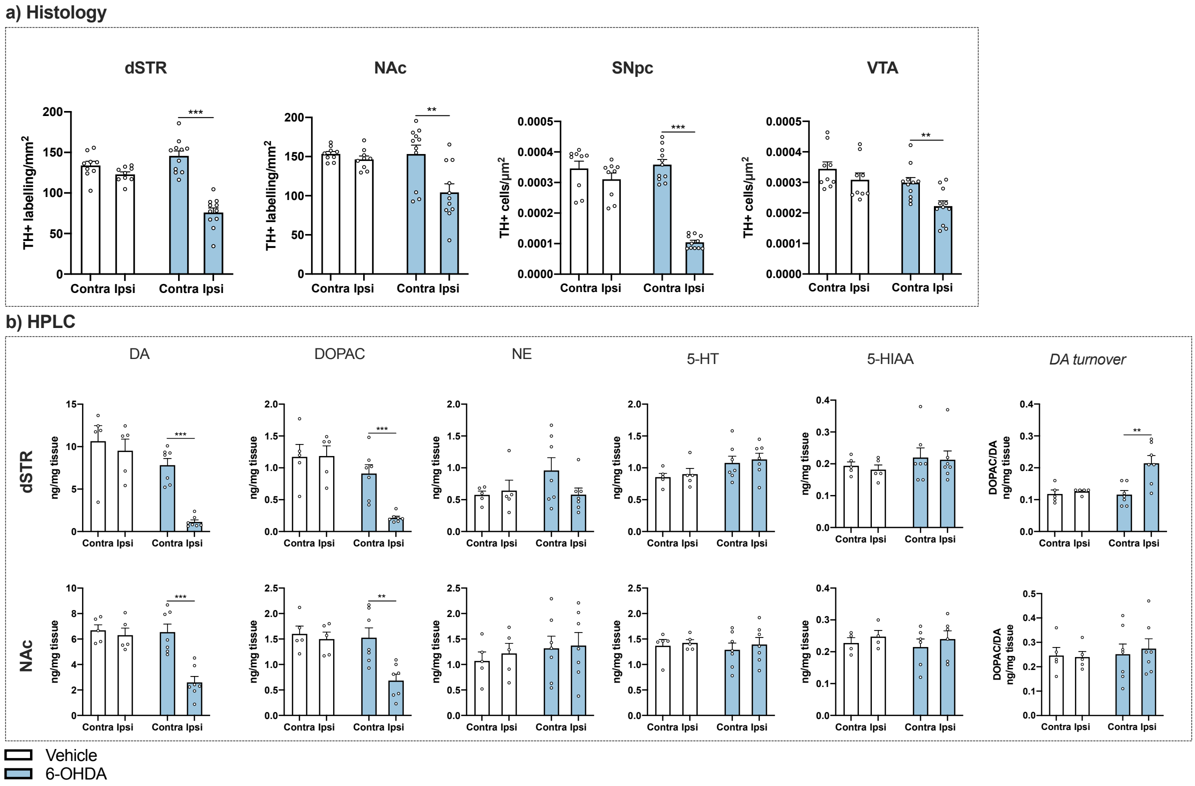

Figure S1. Raw data relative to main Figure 3. Histological characterization **(a)** of intrastriatal 6-OHDA lesions. TH+ labelling was measured in the dorsal striatum and NAc, and TH+ cell counts were performed in the SNpc and VTA. For all regions, significant differences were found between contra- and ipsilateral sides for the 6-OHDA group. Neurochemical analysis **(b)** using HPLC quantification combined with electrochemical detection, showing the dorsal striatum and NAc monoamine profile. Results showed a significant decrease of DA and its metabolite DOPAC for both regions, and an enhancement of DA turnover in the dorsal striatum, when comparing the contra- and ipsilateral sides of the 6-OHDA group. No differences were found for the other monoamines and its derivates (NE, 5-HT, and 5-HIAA) (Student's t test between contra and ipsilateral sides for each group; statistical summary in Table S2; data are presented as mean ± SEM). For histological analysis vehicle n = 9, 6-OHDA n = 11; for HPLC vehicle = 4-5, 6-OHDA n = 6-7. ** $p < 0.01$, *** $p < 0.001$. Abbreviations: dSTR, dorsal striatum; DA, Dopamine; DOPAC, 3,4-Dihydroxyphenylacetic acid; NAc, nucleus accumbens; NE, Norepinephrine; SNpc, substantia nigra pars compacta; TH, tyrosine hydroxylase; VTA, ventral tegmental area; 5-HIAA, 5-Hydroxyindoleacetic acid; 5-HT, serotonin; 6-OHDA, 6-hydroxydopamine.

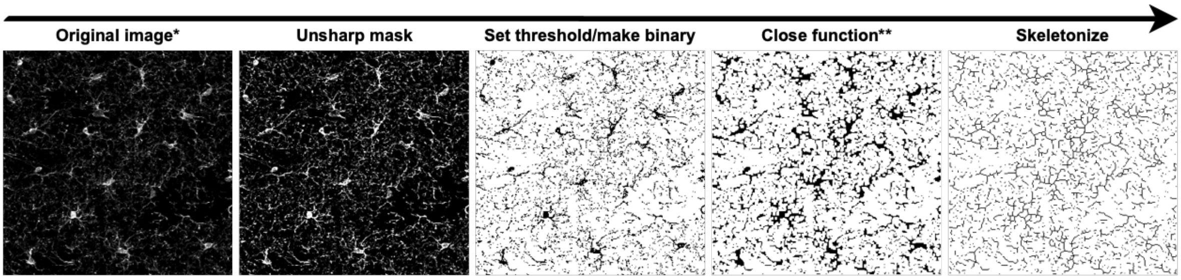

*Representative section (supposed to be applied to the whole image)

**Joins pixels less than 2 pixels apart

Analyze skeleton function

# Branches	# Junctions	# End-point voxels	# Junction voxels	# Slab voxels	Average Branch Length	# Triple points	# Quadruple points	Maximum Branch Length
1	0	2	0	2	3.045	0	0	3.045
0	0	1	0	0	0.000	0	0	0.000
1	0	2	0	12	12.318	0	0	12.318
0	0	1	0	0	0.000	0	0	0.000
0	0	1	0	0	0.000	0	0	0.000
0	0	1	0	0	0.000	0	0	0.000
3	1	3	3	2	2.076	1	0	2.716
0	0	1	0	0	0.000	0	0	0.000
1	0	2	0	0	1.125	0	0	1.125
0	0	1	0	0	0.000	0	0	0.000
1	0	2	0	8	8.477	0	0	8.477
0	0	1	0	0	0.000	0	0	0.000
1	0	2	0	0	0.795	0	0	0.795
1	0	2	0	5	5.102	0	0	5.102
1	0	2	0	0	0.795	0	0	0.795
1	0	2	0	0	0.795	0	0	0.795
1	0	2	0	5	5.432	0	0	5.432
0	0	1	0	0	0.000	0	0	0.000
1	0	2	0	0	0.795	0	0	0.795
3	1	3	4	5	3.466	1	0	3.841
4	1	4	5	15	5.151	0	1	14.046
0	0	1	0	0	0.000	0	0	0.000
0	0	1	0	0	0.000	0	0	0.000
1	0	2	0	0	0.795	0	0	0.795
0	0	1	0	0	0.000	0	0	0.000

Figure S2. Microglia morphology quantification workflow. Photomicrographs (20x magnification) were analyzed using a semi-automated workflow on FIJI. Microglial branching was assessed with the skeletonize tool after the application of an unsharp mask and the close function. Average branch length ≤ 2 were removed from the analysis (considered as noise). Total branch length (Average branch length x Branches) was normalized by the number of microglial cells (IBA1+ cells) within each image.

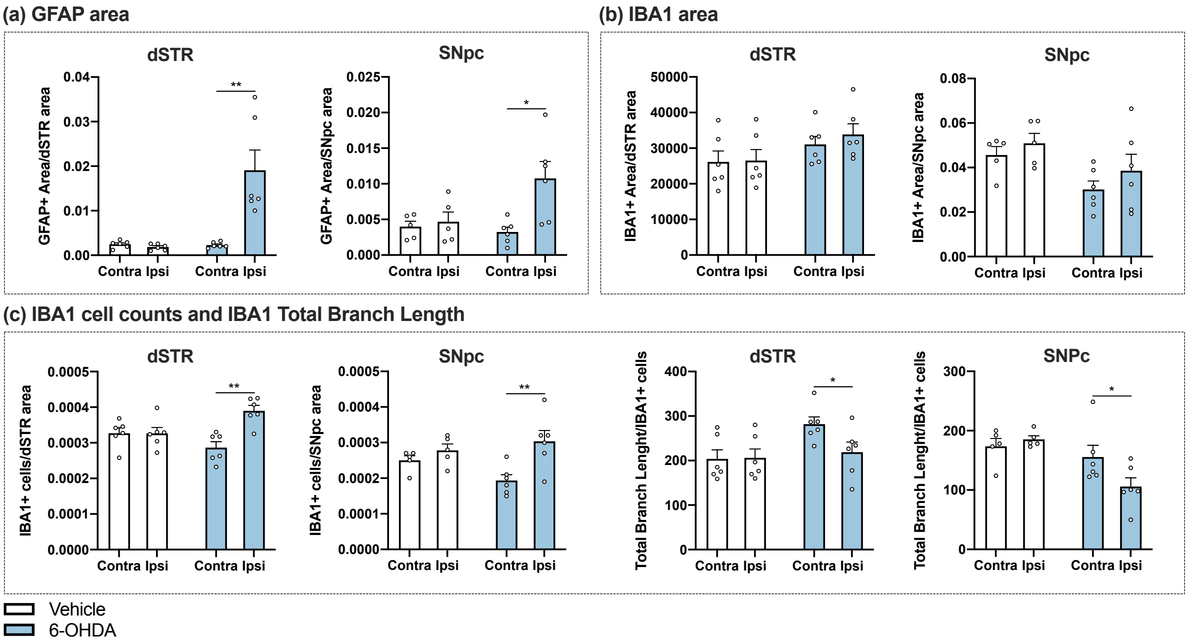

Figure S3. Raw data relative to main Figure 4 and Figure 5. Quantification of area coverage by GFAP+ staining **(a)** showed a significant astrocytic reaction between the contra- and ipsilateral sides for the 6-OHDA group. No differences were found between the contra- and ipsilateral sides of both groups after quantification of area coverage by IBA1+ staining in the dorsal striatum and SNpc **(b)**. A significant increase of IBA1+ cells, as wells as, a significant decrease of IBA1+ total branch length **(c)** in the ipsilateral areas of the 6-OHDA group was observed, indicating the presence of proliferative and activated microglia (Student's t-test; statistical summary in Table S2; data are presented as mean ± SEM). Vehicle n = 4-5, 6-OHDA n = 6-7 (4 slices/animal). * $p < 0.05$, ** $p < 0.01$. Abbreviations: dSTR, dorsal striatum; GFAP, glial fibrillary acidic protein; SNpc, substantia nigra pars compacta; 6-OHDA, 6-hydroxydopamine.

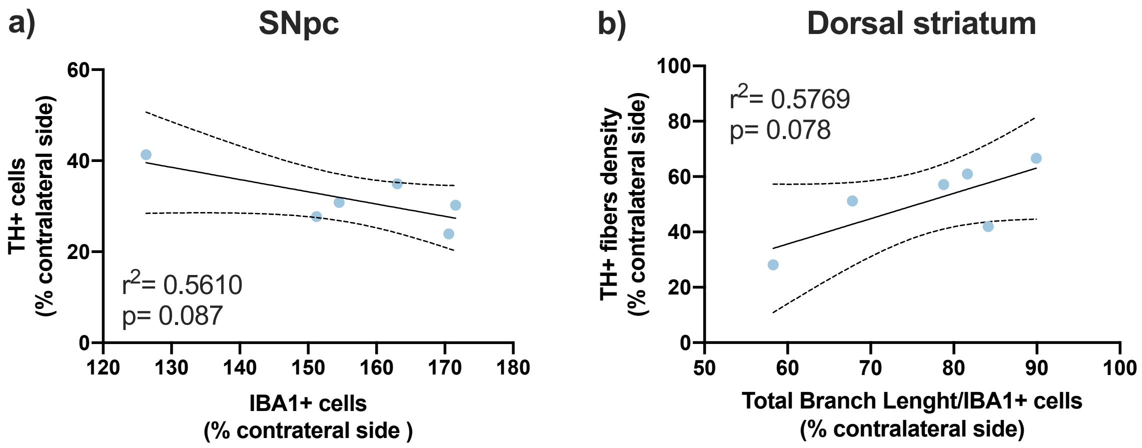

Figure S4. Correlation between dopaminergic loss and microglial cells. Scatter plots representing the correlation between TH+ cells and IBA1+ cells in SNpc **(a)** and TH+ fibers density with microglial branching (total branch length/IBA1+ cells) **(b)** for 6-OHDA-lesioned mice (Pearson correlation; statistical summary in Table S2). 6-OHDA n=6. Abbreviations: SNpc, substantia nigra pars compacta; TH, tyrosine hydroxylase; 6-OHDA, 6-hydroxydopamine.

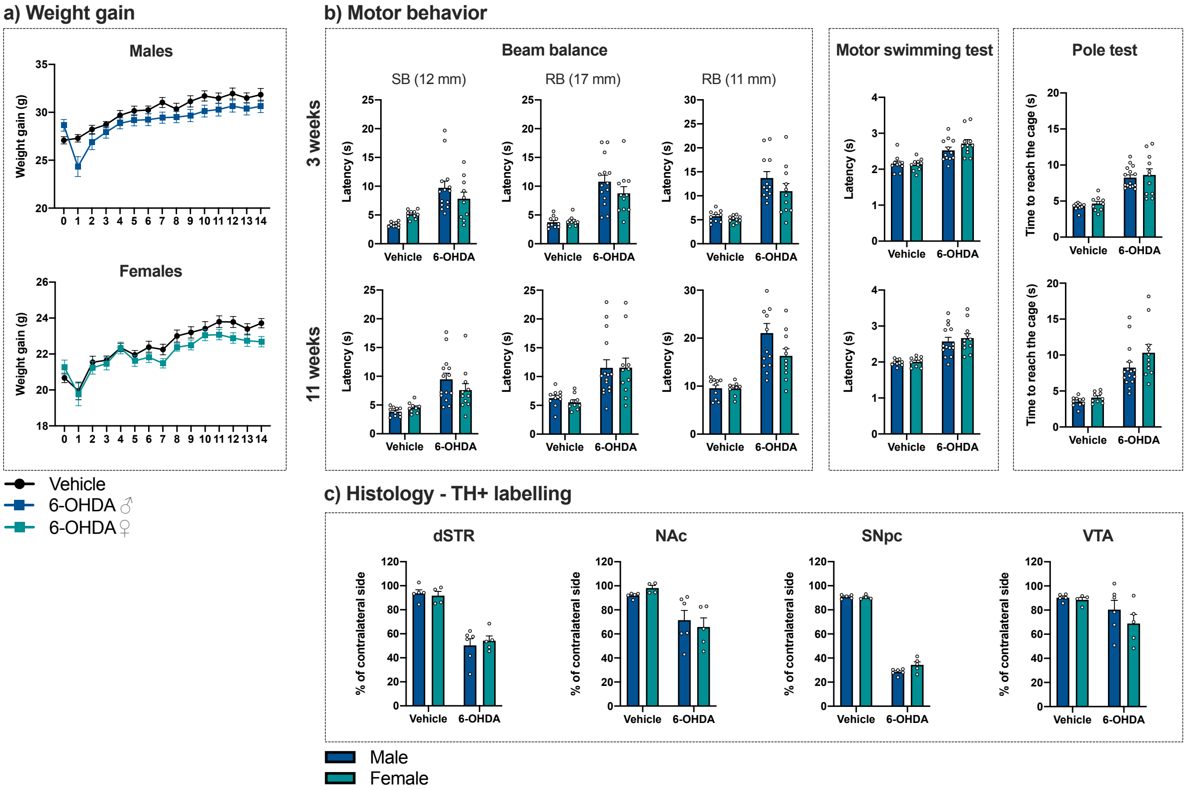

Figure S5. Impact of sex in behavioral and histological outcomes after 6-OHDA intrastriatal lesion. No differences were found in the weight gain for both males **(a)** and females **(b)** after 6-OHDA injections, when compared to the vehicle group, until de the end of the in vivo experiments (14 weeks) (Repeated measures ANOVA; statistical summary in Table S2; data are presented as mean ± SEM). No impact of sex or interaction between sex and group were significative in the motor behavior **(b)** and histological analysis **(c)** (Two-way ANOVA; statistical summary in Table S2; data are presented as mean ± SEM). The only source of variation was the group, i.e. the 6-OHDA lesion (these differences are not represented on the graphs, but are summarized in Table S2). For weight gain and motor behavior: vehicle$_{males}$ = 10, 6-OHDA$_{males}$ = 15, vehicle$_{females}$ = 9-10, 6-OHDAfe$_{males}$ = 11; for histological analysis vehicle$_{males}$ = 5, 6-OHDA$_{males}$ = 6, vehicle$_{females}$ = 4, 6-OHDAfe$_{males}$ = 5. Abbreviations: 6-OHDA, 6-hydroxydopamine.

Table S1. Statistical reports of all performed analysis in main Figures

Measure		Statistical report: 3 weeks	Statistical report: 11 weeks
**Beam balance** (Figure 1a) *Latency (s)*	Square beam (12 mm)	$t_{(42)}$=5.03, p<0.001, d=1.58	$t_{(42)}$=4.83, p<0.001, d=1.56
	Round beam (17 mm)	$t_{(43)}$=6.48, p<0.001, d=2.04	$t_{(42)}$=4.44, p<0.001, d=1.43
	Round beam (11 mm)	$t_{(41)}$=6.10, p<0.001, d=1.93	$t_{(42)}$=5.88, p<0.001, d=1.90
**Motor swimming test** (Figure 1b) *Latency (s)*		$t_{(41)}$=5.51, p<0.001, d=1.71	$t_{(42)}$=6.31, p<0.001, d=2.04
**Pole test** (Figure 1c) *Time to reach the cage (s)*		$t_{(43)}$=7.92, p<0.001, d=2.54	$t_{(43)}$=6.67, p<0.001, d=2.16
**Cylinder test** (Figure 1d) *Left paw contacts as % of total*		$t_{(17)}$=4.52, p<0.001, d=2.10	$t_{(15)}$=5.33, p<0.001, d=2.54
**Open arena** (Figure 1e)	Nr. of squares traveled	U=45, p<0.001	U=73.50, p<0.001
	Gait quality	U=120, p<0.001	U=171, p<0.05
**Rotameter test** (Figure 1f) *Net rotation*		U=0, p<0.001	
**Open field** (Figure 2a) *Time in center*		$t_{(43)}$=0.89, p=0.3785	
**Elevated-plus maze** (Figure 2b) *Time in opened arms*		$t_{(43)}$=1.44, p=0.156	
**Forced swim test** (Figure 2c) *Immobility time*		$t_{(39)}$=2.30, p<0.05, d=0.75	
**Splash-sucrose test** (Figure 2d) *Time grooming*		$t_{(41)}$=2.46, p<0.05, d=0.78	
**TH+ labelling** (Figure 3c/d)	**dSTR**	$t_{(18)}$=9.58, p<0.001, d=4.43	
	**NAc**	$t_{(18)}$=4.25, p<0.001, d=2.00	
**TH+ labelling** (Figure 3e/f)	**SNpc**	$t_{(18)}$=33.76, p<0.001, d=15.85	
	**VTA**	$t_{(18)}$=2.33, p<0.05, d=1.10	
**Neurochemical analysis** (Figure 3g/h)		**dSTR**	**NAc**
	**DA**	$t_{(10)}$=6.08, p<0.001, d=3.22	$t_{(10)}$=4.20, p<0.01, d=2.46
	**DOPAC**	$t_{(10)}$=7.76, p<0.001, d=4.21	$t_{(10)}$=3.25, p<0.01, d=2.00
	**NE**	$t_{(10)}$=1.63, p=0.135	$t_{(10)}$=0.48, p=0.635
	**5-HT**	$t_{(10)}$=0.07, p=0.946	$t_{(10)}$=0.19, p=0.856
	**5-HIAA**	$t_{(10)}$=0.48, p=0.644	$t_{(8)}$=0.53, p=0.609
**DA turnover (Figure 3i) dSTR**		$t_{(10)}$=2.40, p<0.05, d=1.51	
**GFAP+ Area** (Figure 4c/d)	**dSTR**	$t_{(10)}$=3.36, p<0.01, d=1.94	
	**SNpc**	$t_{(9)}$=3.07, p<0.05, d=1.95	
**IBA1+ area** (Figure 5c/f)	**dSTR**	$t_{(10)}$=0.89, p=0.396	
	**SNpc**	$t_{(9)}$=0.69, p=0.506	
**IBA1+ cells** (Figure 5d/g)	**dSTR**	$t_{(10)}$=3.51, p<0.01, d=2.03	
	**SNpc**	$t_{(9)}$=4.56, p<0.01, d=2.77	
**IBA1 branch length** (Figure 5e/h)	**dSTR**	$t_{(10)}$=4.08, p<0.01, d=2.36	
	**SNpc**	$t_{(9)}$=3.49, p<0.01, d=2.11	

Table S2. Statistical reports of all performed analysis in supplementary Figures

Measure			Statistical report	
**Correlation** (Figure S2a)<br>*DA neurons with IBA1+ cells*			r2=0.561, p=0.087	
**Correlation** (Figure S2b)<br>*DA fibers with Branch Length*			r2=0.577, p=0.078	
**Weight gain**<br>(Figure S3a)	Males		Time: $F_{(3.7, 86.1)}$=36.41, p<0.001; Group: $F_{(1, 23)}$=2.13, p=0.158<br>Time x Group: $F_{(14, 322)}$=2.98, p<0.001	
	Females		Time: $F_{(2.7, 48.7)}$=37.52, p<0.001; Group: $F_{(1, 18)}$=1.85, p=0.191<br>Time x Group: $F_{(14, 252)}$=1.339, p=0.185	
**Time Point (**Figure S5b)			**3 weeks**	**11 weeks**
**Beam balance**<br>*Latency (s)*	Square beam (12 mm)		Group: $F_{(1, 40)}$=24.47, p<0.001<br>Sex: $F_{(1, 40)}$=0.004, p=0.952<br>Group x Sex: $F_{(1,40)}$=4.07, p=0.051	Group: $F_{(1, 40)}$=22.11, p<0.001<br>Sex: $F_{(1, 40)}$=0.42, p=0.521<br>Group x Sex: $F_{(1,40)}$=1.93, p=0.521
	Round beam (17 mm)		Group: $F_{(1, 41)}$=40.68, p<0.001<br>Sex: $F_{(1, 41)}$=0.96, p=0.333<br>Group x Sex: $F_{(1,41)}$=1.44, p=0.237	Group: $F_{(1, 41)}$=18.81, p<0.001<br>Sex: $F_{(1, 41)}$=0.07, p=0.788<br>Group x Sex: $F_{(1,41)}$=0.09, p=0.772
	Round beam (11 mm)		Group: $F_{(1, 39)}$=37.68, p<0.001<br>Sex: $F_{(1, 39)}$=2.16, p= 0.150<br>Group x Sex: $F_{(1,39)}$=0.92, p=0.343	Group: $F_{(1, 40)}$=34.83, p<0.001<br>Sex: $F_{(1, 40)}$=2.41, p=0.128<br>Group x Sex: $F_{(1,40)}$=2.28, p=0.139
**Motor swimming test**<br>*Latency (s)*			Group: $F_{(1, 39)}$=31.49, p<0.001<br>Sex: $F_{(1, 39)}$=0.94, p=0.340<br>Group x Sex: $F_{(1,39)}$=1.65, p=0.210	Group: $F_{(1, 40)}$=38.71, p<0.001<br>Sex: $F_{(1, 40)}$=0.32, p=0.574<br>Group x Sex: $F_{(1,40)}$=0.12, p=0.734
**Pole test**<br>*Time to reach the cage (s)*			Group: $F_{(1, 42)}$=62.07, p<0.001<br>Sex: $F_{(1, 42)}$=0.57, p=0.456<br>Group x Sex: $F_{(1,42)}$=0.002, p=0.968	Group: $F_{(1, 41)}$=48.77, p<0.001<br>Sex: $F_{(1, 41)}$=2.82, p=0.100<br>Group x Sex: $F_{(1,41)}$=0.90, p=348
**TH+ labelling**<br>(Figure S3c)			**dSTR**	Group: $F_{(1, 16)}$=82.04, p<0.001<br>Sex: $F_{(1, 16)}$=0.04, p=0.847<br>Group x Sex: $F_{(1,16)}$=0.44, p=0.517
			**NAc**	Group: $F_{(1, 16)}$=17.54, p<0.001<br>Sex: $F_{(1, 16)}$=0001, p=0.973<br>Group x Sex: $F_{(1,16)}$=0.90, p=0.358
**TH+ labelling**<br>(Figure S3c)			**SNpc**	Group: $F_{(1, 16)}$=1504, p<0.001<br>Sex: $F_{(1, 16)}$=3.18, p=0.093<br>Group x Sex: $F_{(1,16)}$=4.25, p=0.060
			**VTA**	Group: $F_{(1, 16)}$=5.66 p<0.05<br>Sex: $F_{(1, 16)}$=1.17, p=0.296<br>Group x Sex: $F_{(1,16)}$=0.60, p=0.448
**TH+ labelling**<br>(Figure S3a)	**dSTR**		**Vehicle**	$t_{(16)}$=1.77, p=0.0954
			**6-OHDA**	$t_{(20)}$=8.28, p<0.001
	**NAc**		**Vehicle**	$t_{(16)}$=1.32, p=0.204
			**6-OHDA**	$t_{(20)}$=3.12, p<0.01
**TH+ cells**<br>(Figure S3a)	**SNpc**		**Vehicle**	$t_{(16)}$=1.15, p=0.268
			**6-OHDA**	$t_{(20)}$=14.39, p<0.001
	**VTA**		**Vehicle**	$t_{(16)}$=1.12, p=0.279
			**6-OHDA**	$t_{(20)}$=3.17, p<0.01
**Neurochemical analysis**<br>(Figure S4b)			**dSTR**	**NAc**
	**DA**	**Vehicle**	$t_{(8)}$=0.50, p=0.633	$t_{(8)}$=0.54, p=0.601
		**6-OHDA**	$t_{(12)}$=8.19, p<0.001	$t_{(12)}$=5.11, p<0.001
	**DOPAC**	**Vehicle**	$t_{(8)}$=0.06, p=0.957	$t_{(8)}$=0.50, p=0.631

		**6-OHDA**	$t_{(12)}$=4.90, p<0.001	$t_{(12)}$=3.66, p<0.01
	**NE**	**Vehicle**	$t_{(8)}$=0.38, p=0.715	$t_{(8)}$=0.56, p=0.594
		**6-OHDA**	$t_{(12)}$=1.68, p=0.119	$t_{(12)}$=0.16, p=0.875
	**5-HT**	**Vehicle**	$t_{(8)}$=0.42, p=0.685	$t_{(8)}$=0.41, p=0.694
		**6-OHDA**	$t_{(12)}$=0.39, p=0.704	$t_{(12)}$=0.55, p=0.591
	**5-HIAA**	**Vehicle**	$t_{(8)}$=0.63, p=0.545	$t_{(6)}$=0.80, p=0.461
		**6-OHDA**	$t_{(12)}$=0.18, p=0.864	$t_{(12)}$=0.69, p=0.506
	**DOPAC/DA**	**Vehicle**	$t_{(8)}$=0.61, p=0.557	$t_{(8)}$=0.15, p=0.884
		**6-OHDA**	$t_{(12)}$=3.66, p<0.01	$t_{(12)}$=0.39, p=0.701
**Glial reaction analysis** (Figure S5)			**dSTR**	**SNpc**
	**GFAP+ area**	**Vehicle**	$t_{(10)}$=1.41, p=0.189	$t_{(8)}$=0.45, p=0.663
		**6-OHDA**	$t_{(10)}$=3.71, p<0.01	$t_{(10)}$=3.06, p<0.05
	**IBA1+ area**	**Vehicle**	$t_{(10)}$=0.09, p=0.933	$t_{(8)}$=0.89, p=0.397
		**6-OHDA**	$t_{(10)}$=0.75, p=0.469	$t_{(10)}$=1.01, p=0.339
	**IBA1+ cells**	**Vehicle**	$t_{(10)}$=0.02, p=0.983	$t_{(8)}$=1.26, p=0.243
		**6-OHDA**	$t_{(10)}$=4.58, p<0.01	$t_{(10)}$=3.17, p<0.01
	**IBA1 Total Branch Length**	**Vehicle**	$t_{(10)}$=0.08, p=0.936	$t_{(8)}$=0.81, p=0.444
		**6-OHDA**	$t_{(10)}$=2.26, p<0.05	$t_{(10)}$=2.03, p<0.05

Table S3. Perioperative health assessment and procedures after 6-OHDA injection in mice (adapted from [1])

Type	Identification	Treatment
General health	Animal's behavior into the home cage: interaction with the environment, cage mates and nest building	-
Dehydration	Reduced skin turgor; recessed eyes and fuzzy fur	Fluid replacement through subcutaneous or intraperitoneal administration of sterile saline (0.9%)
Hypothermia	Body temperature below 36.5°C; animals are cool to the touch	Increase housing room temperature; administration of warm fluids (e.g., warm sterile saline injections)
Aphagia and adipsia	Measurement of food and water consumption; Body weight monitoring	Easy access to palatable food supplementation; hydration complemented with glucose solution
Penile prolapse	Swollen and distended penis	Lubrification; soft bed; local analgesia
Spontaneous turning behavior	Animals spontaneously rotate to the ipsilateral side (unilateral lesion)	Animals adapt to rotational movement

Table S4. Confocal microscopy for glial reaction: acquisition paramaters

Region	Antigen	PMT Voltage (V)	Laser Transmissivity (%)	Emission Wavelength (nm)	Detection Wavelength (nm)	Detector Gain	Detector Offset (%)
**SNpc Mosaics**	DAPI	410	1.01	461	430-470	1.25	8.0
	TH	430	1.80	520	500-540	1.0	10.0
	GFAP	600	6.49	618	570-620	1.0	11.0
	IBA-1	570	4.88	671	650-750	1.0	10.0
**Striatum Images**	DAPI	410	1.30	461	430-470	1.0	12.0
	TH	460	2.0	520	500-540	1.0	12.0
	GFAP	650	7.0	618	570-620	1.0	14.0
	IBA-1	595	5.0	671	650-750	1.0	13.0

References

1. Masini, D.; Plewnia, C.; Bertho, M.; Scalbert, N.; Caggiano, V.; Fisone, G. A Guide to the Generation of a 6-Hydroxydopamine Mouse Model of Parkinson's Disease for the Study of Non-Motor Symptoms. *Biomedicines* **2021**, *9*, 598, doi:10.3390/biomedicines9060598.
